# Bis[3-ethyl-4-(4-methoxy­phen­yl)-5-(2-pyrid­yl)-4*H*-1,2,4-triazole-κ^2^ *N* ^1^,*N* ^5^]bis­(perchlorato-κO)copper(II) acetonitrile disolvate

**DOI:** 10.1107/S1600536808012026

**Published:** 2008-04-30

**Authors:** Liaocheng Huang, Zuoxiang Wang, Xiaoming Zhang, Pingfeng Wu

**Affiliations:** a Ordered Matter Science Research Center, Southeast University, Nanjing 210096, People’s Republic of China

## Abstract

In the title compound, [Cu(ClO_4_)_2_(C_16_H_16_N_4_O)_2_]·2CH_3_CN, the Cu^II^ atom, located on an inversion center, is in a tetra­gonally distorted octa­hedral environment, coordinated by four N atoms of two bidentate 3-ethyl-4-(4-methoxy­phen­yl)-5-(2-pyrid­yl)-4H-1,2,4-triazole ligands in equatorial positions and by the O atoms of two perchlorate groups in axial positions. The long axial Cu—O bond of 2.4743 (17) Å is the result of the Jahn–Teller effect.

## Related literature

For related literature, see: Bencini *et al.* (1987 ▶); Garcia *et al.* (1997 ▶); Kahn & Martinez (1998 ▶); Klingele *et al.* (2005 ▶, 2006 ▶); Koningsbruggen (2004 ▶); Koningsbruggen *et al.* (1995 ▶); Lavrenova & Larionov (1998 ▶); Matouzenko *et al.* (2004 ▶); Moliner *et al.* (1998 ▶, 2001 ▶); Wang *et al.* (2005 ▶); Zhou *et al.*, (2006*a* ▶,*b* ▶).
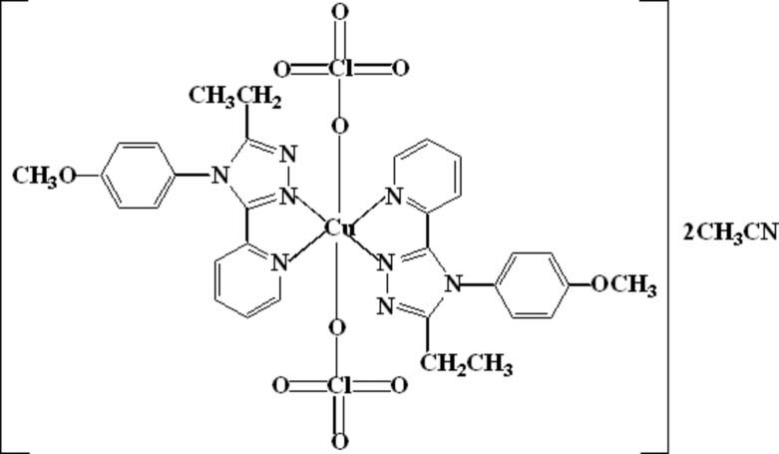


## Experimental

### 

#### Crystal data

[Cu(ClO_4_)_2_(C_16_H_16_N_4_O)_2_]·2C_2_H_3_N
*M* *_r_* = 905.21
Triclinic, 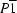

*a* = 8.3286 (11) Å
*b* = 9.1266 (14) Å
*c* = 14.225 (2) Å
α = 100.516 (7)°
β = 101.067 (4)°
γ = 98.780 (4)°
*V* = 1023.4 (3) Å^3^
*Z* = 1
Mo *K*α radiation
μ = 0.73 mm^−1^
*T* = 293 (2) K
0.20 × 0.20 × 0.20 mm

#### Data collection

Rigaku Mercury2 diffractometer
Absorption correction: multi-scan (*CrystalClear*; Rigaku/MSC, 2005 ▶) *T*_min_ = 0.864, *T*_max_ = 0.867
8302 measured reflections
3565 independent reflections
3170 reflections with *I* > 2σ(*I*)
*R*_int_ = 0.031

#### Refinement

*R*[*F* ^2^ > 2σ(*F* ^2^)] = 0.039
*wR*(*F* ^2^) = 0.120
*S* = 1.16
3565 reflections
271 parameters
H-atom parameters constrained
Δρ_max_ = 0.46 e Å^−3^
Δρ_min_ = −0.54 e Å^−3^

### 

Data collection: *CrystalClear* (Rigaku/MSC, 2005 ▶); cell refinement: *CrystalClear*; data reduction: *CrystalClear*; program(s) used to solve structure: *SHELXS97* (Sheldrick, 2008 ▶); program(s) used to refine structure: *SHELXL97* (Sheldrick, 2008 ▶); molecular graphics: *SHELXTL/PC* (Sheldrick, 2008 ▶); software used to prepare material for publication: *SHELXTL/PC*.

## Supplementary Material

Crystal structure: contains datablocks I, global. DOI: 10.1107/S1600536808012026/gk2140sup1.cif

Structure factors: contains datablocks I. DOI: 10.1107/S1600536808012026/gk2140Isup2.hkl

Additional supplementary materials:  crystallographic information; 3D view; checkCIF report

## Figures and Tables

**Table:** 

Cu1—N2	1.9892 (16)
Cu1—N1	2.0261 (18)
Cu1—O2	2.4743 (17)

**Table:** 

N2^i^—Cu1—N2	180
N2^i^—Cu1—N1	99.31 (7)
N2—Cu1—N1	80.69 (7)
N1—Cu1—N1^i^	180
N2^i^—Cu1—O2	92.07 (7)
N2—Cu1—O2	87.93 (7)
N1—Cu1—O2	92.61 (7)
N1^i^—Cu1—O2	87.39 (7)

Symmetry code: (i) 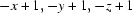.

**Fig. 1.**
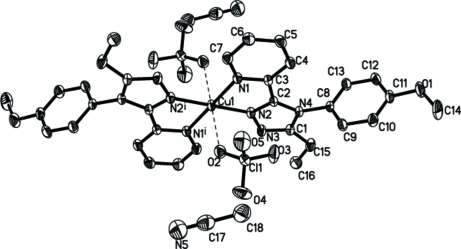
The molecular structure of the title compound with displacement ellipsoids shown at the 30% probability level. Symmetry code (i):-x + 1,-y + 1,-z + 1

## supplementary crystallographic information

### Comment

1,2,4-Triazole derivatives can be used as bridging ligands in transition metal coordination chemistry (Bencini *et al.*, 1987; Koningsbruggen *et al.*, 1995; Moliner *et al.*, 1998, 2001; Klingele *et al.*, 2005, 2006). Some spin-crossover complexes of 1,2,4-triazoles with iron(II) salts have been reported with potential applications as molecular-based memory devices, displays and optical switches (Garcia *et al.*, 1997; Lavrenova & Larionov, 1998; Kahn & Martinez, 1998; Koningsbruggen 2004; Matouzenko *et al.*, 2004). Recently we have reported synthesis of some new 3,4-disubstituted-5-(2-pyridyl)-1,2,4-triazole ligands and crystal structures of their transition-metal complexes (Wang *et al.*, 2005; Zhou *et al.*, 2006a,b). Here we report the crystal structure of the title compound (Fig. 1). The Cu^II^ atom is located on an inversion center. It shows a tetragonally distorted octahedral coordination geometry and is coordinated by two bis-chelating 3-ethyl-4-(4-methoxyphenyl)-5-(2-pyridyl)-1,2,4-triazole ligands with the CuN_2_N'_2_ chromophore in the equatorial plane and two O atoms of two perchlorate groups in axial positions.

### Experimental

The title compound was prepared by the reaction of 3-ethyl-4-(4-methoxyphenyl)-5-(2-pyridyl)-1,2,4-triazole with copper(II) perchlorate in acetonitrile. To a warm solution of 3-ethyl-4-(4-methoxyphenyl)-5-(2-pyridyl)-1,2,4-triazole (1.120 g, 4.0 mmol) in 20 ml acetonitrile, copper(II) perchlorate (0.525 g, 2.0 mmol) was added. After several days blue single crystals suitable for X-ray analysis were collected.

### Refinement

All H atoms were located in a difference Fourier map and allowed to ride on their parent atoms at distances of 0.93 Å(C—H aromatic), 0.97 Å(C—H methylene) and 0.96 Å(C—H methyl), and with *U*_iso_(H) values of 1.2–1.5 times *U*_eq_ of the parent atoms.

### Crystal data

**Table:**

[Cu(ClO_4_)_2_(C_16_H_16_N_4_O)_2_]·2C_2_H_3_N	*Z* = 1
*M**_r_* = 905.21	*F*_000_ = 467
Triclinic, *P*1	*D*_x_ = 1.469 Mg m^−^^3^
Hall symbol: -P 1	Mo *K*α radiation λ = 0.71073 Å
*a* = 8.3286 (11) Å	Cell parameters from 2714 reflections
*b* = 9.1266 (14) Å	θ = 3.1–27.5º
*c* = 14.225 (2) Å	µ = 0.73 mm^−^^1^
α = 100.516 (7)º	*T* = 293 (2) K
β = 101.067 (4)º	Prism, blue
γ = 98.780 (4)º	0.20 × 0.20 × 0.20 mm
*V* = 1023.4 (3) Å^3^	

### Data collection

**Table:**

Rigaku Mercury2 diffractometer	3565 independent reflections
Radiation source: fine-focus sealed tube	3170 reflections with *I* > 2σ(*I*)
Monochromator: graphite	*R*_int_ = 0.031
Detector resolution: 13.6612 pixels mm^-1^	θ_max_ = 25.0º
*T* = 293(2) K	θ_min_ = 2.3º
ω scans	*h* = −9→9
Absorption correction: multi-scan(CrystalClear; Rigaku/MSC, 2005)	*k* = −10→10
*T*_min_ = 0.864, *T*_max_ = 0.867	*l* = −16→16
8302 measured reflections	

### Refinement

**Table:**

Refinement on *F*^2^	Secondary atom site location: difference Fourier map
Least-squares matrix: full	Hydrogen site location: inferred from neighbouring sites
*R*[*F*^2^ > 2σ(*F*^2^)] = 0.039	H-atom parameters constrained
*wR*(*F*^2^) = 0.120	*w* = 1/[σ^2^(*F*_o_^2^) + (0.0739*P*)^2^] where *P* = (*F*_o_^2^ + 2*F*_c_^2^)/3
*S* = 1.16	(Δ/σ)_max_ < 0.001
3565 reflections	Δρ_max_ = 0.46 e Å^−^^3^
271 parameters	Δρ_min_ = −0.54 e Å^−^^3^
Primary atom site location: structure-invariant direct methods	Extinction correction: none

### Special details

**Table:**

Geometry. All e.s.d.'s (except the e.s.d. in the dihedral angle between two l.s. planes) are estimated using the full covariance matrix. The cell e.s.d.'s are taken into account individually in the estimation of e.s.d.'s in distances, angles and torsion angles; correlations between e.s.d.'s in cell parameters are only used when they are defined by crystal symmetry. An approximate (isotropic) treatment of cell e.s.d.'s is used for estimating e.s.d.'s involving l.s. planes.
Refinement. Refinement of *F*^2^ against ALL reflections. The weighted *R*-factor *wR* and goodness of fit *S* are based on *F*^2^, conventional *R*-factors *R* are based on *F*, with *F* set to zero for negative *F*^2^. The threshold expression of *F*^2^ > σ(*F*^2^) is used only for calculating *R*-factors(gt) *etc*. and is not relevant to the choice of reflections for refinement. *R*-factors based on *F*^2^ are statistically about twice as large as those based on *F*, and *R*- factors based on ALL data will be even larger.

### Fractional atomic coordinates and isotropic or equivalent isotropic displacement parameters (Å^2^)

**Table:**

	*x*	*y*	*z*	*U*_iso_*/*U*_eq_	
Cu1	0.5000	0.5000	0.5000	0.03204 (16)	
Cl1	0.54072 (7)	0.50192 (6)	0.25312 (4)	0.04015 (18)	
O1	−0.0616 (2)	−0.3362 (2)	0.02788 (13)	0.0619 (5)	
O2	0.6082 (2)	0.5545 (2)	0.35718 (12)	0.0508 (4)	
O3	0.4928 (4)	0.3415 (2)	0.23080 (16)	0.0795 (7)	
O4	0.6656 (3)	0.5460 (3)	0.20330 (15)	0.0827 (8)	
O5	0.4003 (3)	0.5667 (3)	0.2258 (2)	0.0942 (8)	
N1	0.2619 (2)	0.43579 (19)	0.42008 (13)	0.0325 (4)	
N2	0.4977 (2)	0.28147 (18)	0.44924 (13)	0.0329 (4)	
N3	0.6125 (2)	0.1869 (2)	0.45117 (13)	0.0370 (4)	
N4	0.3825 (2)	0.07416 (18)	0.34047 (12)	0.0315 (4)	
N5	0.7131 (5)	0.9957 (4)	0.1193 (3)	0.1093 (12)	
C1	0.5401 (3)	0.0628 (2)	0.38456 (15)	0.0354 (5)	
C2	0.3617 (3)	0.2129 (2)	0.38257 (14)	0.0302 (5)	
C3	0.2210 (3)	0.2907 (2)	0.36606 (15)	0.0305 (4)	
C4	0.0646 (3)	0.2305 (3)	0.30709 (16)	0.0401 (5)	
H1	0.0398	0.1314	0.2703	0.048*	
C5	−0.0554 (3)	0.3207 (3)	0.30364 (18)	0.0455 (6)	
H2	−0.1622	0.2825	0.2647	0.055*	
C6	−0.0147 (3)	0.4656 (3)	0.35806 (19)	0.0470 (6)	
H3	−0.0940	0.5272	0.3566	0.056*	
C7	0.1451 (3)	0.5213 (2)	0.41562 (17)	0.0410 (5)	
H4	0.1716	0.6208	0.4521	0.049*	
C8	0.2691 (3)	−0.0342 (2)	0.25825 (15)	0.0317 (5)	
C9	0.2728 (3)	−0.0173 (3)	0.16435 (16)	0.0419 (6)	
H5	0.3484	0.0613	0.1543	0.050*	
C10	0.1638 (3)	−0.1176 (3)	0.08535 (17)	0.0467 (6)	
H10A	0.1665	−0.1073	0.0218	0.056*	
C11	0.0513 (3)	−0.2327 (3)	0.10070 (17)	0.0425 (6)	
C12	0.0493 (3)	−0.2497 (3)	0.19559 (19)	0.0491 (6)	
H12A	−0.0261	−0.3281	0.2059	0.059*	
C13	0.1592 (3)	−0.1503 (3)	0.27446 (17)	0.0433 (6)	
H13A	0.1589	−0.1618	0.3381	0.052*	
C14	−0.0529 (5)	−0.3328 (4)	−0.0708 (2)	0.0801 (11)	
H14A	−0.1345	−0.4146	−0.1148	0.120*	
H14B	0.0564	−0.3439	−0.0795	0.120*	
H14C	−0.0748	−0.2377	−0.0845	0.120*	
C15	0.6135 (3)	−0.0739 (3)	0.3591 (2)	0.0476 (6)	
H15A	0.6122	−0.0932	0.2896	0.057*	
H15B	0.5440	−0.1609	0.3710	0.057*	
C16	0.7893 (3)	−0.0596 (3)	0.4159 (2)	0.0558 (7)	
H16A	0.8271	−0.1528	0.3978	0.084*	
H16B	0.7922	−0.0392	0.4849	0.084*	
H16C	0.8606	0.0221	0.4013	0.084*	
C17	0.6382 (5)	0.8821 (5)	0.0784 (3)	0.0736 (9)	
C18	0.5389 (5)	0.7344 (4)	0.0255 (3)	0.0826 (10)	
H18A	0.4634	0.6987	0.0633	0.124*	
H18B	0.6113	0.6636	0.0150	0.124*	
H18C	0.4766	0.7434	−0.0367	0.124*	

### Atomic displacement parameters (Å^2^)

**Table:**

	*U*^11^	*U*^22^	*U*^33^	*U*^12^	*U*^13^	*U*^23^
Cu1	0.0291 (2)	0.0242 (2)	0.0355 (2)	0.00483 (15)	−0.00289 (16)	−0.00093 (16)
Cl1	0.0357 (3)	0.0382 (3)	0.0421 (3)	0.0009 (2)	0.0040 (2)	0.0079 (3)
O1	0.0567 (12)	0.0558 (11)	0.0491 (11)	−0.0125 (9)	−0.0083 (9)	−0.0088 (9)
O2	0.0526 (11)	0.0506 (10)	0.0418 (9)	0.0000 (8)	0.0062 (8)	0.0047 (8)
O3	0.135 (2)	0.0369 (10)	0.0564 (12)	−0.0054 (12)	0.0259 (13)	−0.0023 (9)
O4	0.0610 (14)	0.126 (2)	0.0611 (12)	−0.0114 (13)	0.0182 (11)	0.0415 (14)
O5	0.0570 (14)	0.0994 (18)	0.1069 (19)	0.0306 (13)	−0.0222 (13)	0.0044 (16)
N1	0.0316 (9)	0.0288 (9)	0.0332 (9)	0.0056 (7)	0.0016 (7)	0.0032 (7)
N2	0.0332 (10)	0.0277 (9)	0.0323 (9)	0.0052 (7)	−0.0005 (8)	0.0016 (7)
N3	0.0376 (10)	0.0279 (9)	0.0399 (10)	0.0092 (8)	−0.0005 (8)	0.0008 (8)
N4	0.0342 (10)	0.0241 (8)	0.0312 (9)	0.0021 (7)	0.0023 (7)	0.0014 (7)
N5	0.121 (3)	0.095 (3)	0.104 (3)	−0.002 (2)	0.036 (2)	0.009 (2)
C1	0.0390 (12)	0.0282 (11)	0.0342 (11)	0.0045 (9)	0.0036 (9)	0.0016 (9)
C2	0.0335 (11)	0.0241 (9)	0.0294 (10)	0.0017 (8)	0.0044 (9)	0.0027 (8)
C3	0.0315 (11)	0.0270 (10)	0.0290 (10)	0.0018 (8)	0.0023 (8)	0.0041 (8)
C4	0.0364 (12)	0.0352 (12)	0.0407 (12)	0.0016 (10)	0.0008 (10)	0.0012 (10)
C5	0.0300 (12)	0.0494 (14)	0.0483 (14)	0.0028 (10)	−0.0041 (10)	0.0062 (12)
C6	0.0347 (12)	0.0467 (13)	0.0552 (14)	0.0142 (11)	−0.0007 (11)	0.0062 (12)
C7	0.0379 (13)	0.0329 (11)	0.0475 (13)	0.0090 (10)	0.0033 (11)	0.0021 (10)
C8	0.0346 (11)	0.0238 (10)	0.0308 (10)	0.0027 (9)	0.0023 (9)	−0.0011 (8)
C9	0.0464 (13)	0.0348 (12)	0.0366 (12)	−0.0056 (10)	0.0059 (10)	0.0023 (10)
C10	0.0569 (16)	0.0456 (13)	0.0295 (11)	−0.0004 (12)	0.0035 (11)	0.0031 (10)
C11	0.0409 (13)	0.0345 (11)	0.0393 (12)	0.0012 (10)	−0.0031 (10)	−0.0064 (10)
C12	0.0502 (15)	0.0362 (12)	0.0514 (14)	−0.0105 (11)	0.0104 (12)	0.0022 (11)
C13	0.0509 (14)	0.0363 (12)	0.0359 (12)	−0.0048 (11)	0.0089 (11)	0.0028 (10)
C14	0.093 (3)	0.072 (2)	0.0437 (16)	−0.0080 (19)	−0.0183 (16)	−0.0116 (15)
C15	0.0515 (15)	0.0332 (12)	0.0524 (14)	0.0174 (11)	0.0020 (12)	−0.0022 (11)
C16	0.0468 (15)	0.0451 (14)	0.0750 (19)	0.0169 (12)	0.0103 (14)	0.0091 (13)
C17	0.078 (2)	0.085 (2)	0.0633 (19)	0.016 (2)	0.0250 (18)	0.0214 (19)
C18	0.092 (3)	0.087 (3)	0.069 (2)	0.019 (2)	0.0183 (19)	0.0167 (19)

### Geometric parameters (Å, °)

**Table:**

Cu1—N2^i^	1.9892 (16)	C6—C7	1.387 (3)
Cu1—N2	1.9892 (16)	C6—H3	0.9300
Cu1—N1	2.0261 (18)	C7—H4	0.9300
Cu1—N1^i^	2.0261 (18)	C8—C13	1.375 (3)
Cu1—O2	2.4743 (17)	C8—C9	1.378 (3)
Cl1—O5	1.410 (2)	C9—C10	1.380 (3)
Cl1—O4	1.416 (2)	C9—H5	0.9300
Cl1—O3	1.416 (2)	C10—C11	1.376 (3)
Cl1—O2	1.4409 (18)	C10—H10A	0.9300
O1—C11	1.359 (3)	C11—C12	1.389 (4)
O1—C14	1.425 (4)	C12—C13	1.378 (3)
N1—C7	1.336 (3)	C12—H12A	0.9300
N1—C3	1.360 (3)	C13—H13A	0.9300
N2—C2	1.315 (3)	C14—H14A	0.9600
N2—N3	1.382 (2)	C14—H14B	0.9600
N3—C1	1.313 (3)	C14—H14C	0.9600
N4—C2	1.349 (2)	C15—C16	1.504 (4)
N4—C1	1.370 (3)	C15—H15A	0.9700
N4—C8	1.451 (2)	C15—H15B	0.9700
N5—C17	1.116 (5)	C16—H16A	0.9600
C1—C15	1.488 (3)	C16—H16B	0.9600
C2—C3	1.463 (3)	C16—H16C	0.9600
C3—C4	1.377 (3)	C17—C18	1.455 (5)
C4—C5	1.387 (3)	C18—H18A	0.9600
C4—H1	0.9300	C18—H18B	0.9600
C5—C6	1.361 (3)	C18—H18C	0.9600
C5—H2	0.9300		
			
N2^i^—Cu1—N2	180.0	N1—C7—C6	121.7 (2)
N2^i^—Cu1—N1	99.31 (7)	N1—C7—H4	119.1
N2—Cu1—N1	80.69 (7)	C6—C7—H4	119.1
N2^i^—Cu1—N1^i^	80.69 (7)	C13—C8—C9	120.95 (19)
N2—Cu1—N1^i^	99.31 (7)	C13—C8—N4	120.21 (18)
N1—Cu1—N1^i^	180.0	C9—C8—N4	118.83 (18)
N2^i^—Cu1—O2	92.07 (7)	C8—C9—C10	119.6 (2)
N2—Cu1—O2	87.93 (7)	C8—C9—H5	120.2
N1—Cu1—O2	92.61 (7)	C10—C9—H5	120.2
N1^i^—Cu1—O2	87.39 (7)	C11—C10—C9	119.9 (2)
O5—Cl1—O4	110.60 (17)	C11—C10—H10A	120.0
O5—Cl1—O3	109.64 (17)	C9—C10—H10A	120.0
O4—Cl1—O3	109.77 (15)	O1—C11—C10	124.3 (2)
O5—Cl1—O2	109.11 (14)	O1—C11—C12	115.6 (2)
O4—Cl1—O2	108.51 (12)	C10—C11—C12	120.0 (2)
O3—Cl1—O2	109.19 (12)	C13—C12—C11	120.0 (2)
C11—O1—C14	117.6 (2)	C13—C12—H12A	120.0
Cl1—O2—Cu1	131.53 (10)	C11—C12—H12A	120.0
C7—N1—C3	118.35 (18)	C8—C13—C12	119.4 (2)
C7—N1—Cu1	126.54 (14)	C8—C13—H13A	120.3
C3—N1—Cu1	115.11 (14)	C12—C13—H13A	120.3
C2—N2—N3	109.04 (16)	O1—C14—H14A	109.5
C2—N2—Cu1	112.95 (14)	O1—C14—H14B	109.5
N3—N2—Cu1	136.71 (14)	H14A—C14—H14B	109.5
C1—N3—N2	105.81 (18)	O1—C14—H14C	109.5
C2—N4—C1	105.79 (17)	H14A—C14—H14C	109.5
C2—N4—C8	127.23 (18)	H14B—C14—H14C	109.5
C1—N4—C8	126.67 (17)	C1—C15—C16	113.9 (2)
N3—C1—N4	110.41 (18)	C1—C15—H15A	108.8
N3—C1—C15	126.3 (2)	C16—C15—H15A	108.8
N4—C1—C15	123.28 (19)	C1—C15—H15B	108.8
N2—C2—N4	108.94 (18)	C16—C15—H15B	108.8
N2—C2—C3	119.51 (17)	H15A—C15—H15B	107.7
N4—C2—C3	131.54 (18)	C15—C16—H16A	109.5
N1—C3—C4	122.13 (19)	C15—C16—H16B	109.5
N1—C3—C2	110.79 (17)	H16A—C16—H16B	109.5
C4—C3—C2	127.06 (18)	C15—C16—H16C	109.5
C3—C4—C5	118.8 (2)	H16A—C16—H16C	109.5
C3—C4—H1	120.6	H16B—C16—H16C	109.5
C5—C4—H1	120.6	N5—C17—C18	179.4 (5)
C6—C5—C4	119.1 (2)	C17—C18—H18A	109.5
C6—C5—H2	120.4	C17—C18—H18B	109.5
C4—C5—H2	120.4	H18A—C18—H18B	109.5
C5—C6—C7	119.9 (2)	C17—C18—H18C	109.5
C5—C6—H3	120.0	H18A—C18—H18C	109.5
C7—C6—H3	120.0	H18B—C18—H18C	109.5

Symmetry codes: (i) −*x*+1, −*y*+1, −*z*+1.
